# Supplementary material for: Splice-Junction-Based Mapping of Alternative Isoforms in the Human Proteome
Source: Cell Rep. Author manuscript; Available in PMC 2020 Jan 15. (PMC6961840; doi:10.1016/j.celrep.2019.11.026)
Supplement: 2 [file NIHMS1546469-supplement-2.zip › DF1/PXD006675/VentricularSeptum/VentricularSeptum_19_TPM3_AISEELDHALNDMTSG.pdf]

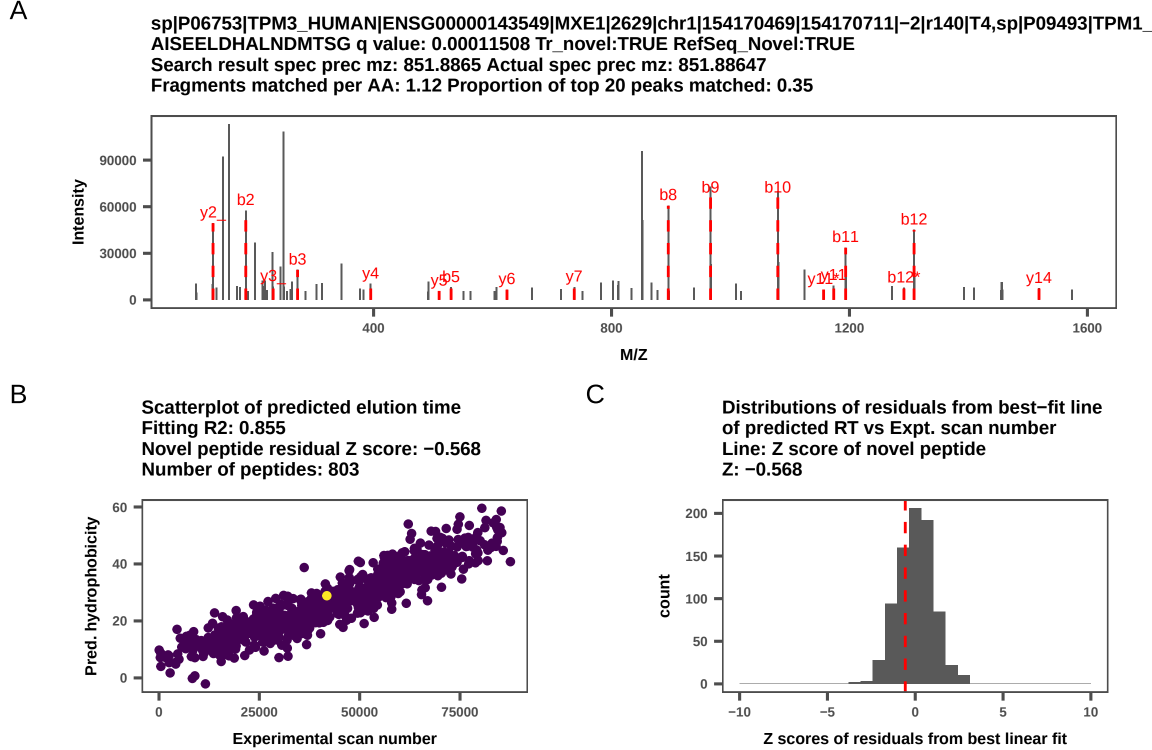
A
sp|P06753|TPM3_HUMAN|ENSG00000143549|MXE1|2629|chr1|154170469|154170711|−2|r140|T4,sp|P09493|TPM1_
AISEELDHALNDMTSG q value: 0.00011508 Tr_novel:TRUE RefSeq_Novel:TRUE
Search result spec prec mz: 851.8865 Actual spec prec mz: 851.88647
Fragments matched per AA: 1.12 Proportion of top 20 peaks matched: 0.35
Intensity
90000
60000
30000
0
y2
b2
b3
y3
y4
y5
b5
y6
y7
b8
b9
b10
b11
y11
b12
b12*
y14
400
800
1200
1600
M/Z
B
Scatterplot of predicted elution time
Fitting R2: 0.855
Novel peptide residual Z score: −0.568
Number of peptides: 803
Pred. hydrophobicity
60
40
20
0
0
25000
50000
75000
Experimental scan number
C
Distributions of residuals from best−fit line
of predicted RT vs Expt. scan number
Line: Z score of novel peptide
Z: −0.568
count
200
150
100
50
0
−10
−5
0
5
10
Z scores of residuals from best linear fit
